# Local Myo9b RhoGAP activity regulates cell motility

**DOI:** 10.1074/jbc.RA120.013623

**Published:** 2020-12-06

**Authors:** Sandra A. Hemkemeyer, Veith Vollmer, Vera Schwarz, Birgit Lohmann, Ulrike Honnert, Muna Taha, Hans-Joachim Schnittler, Martin Bähler

**Affiliations:** 1 Institute of Molecular Cell Biology, Westfalian Wilhelms University Münster, Münster, Germany; 2 Institute of Anatomy & Vascular Biology, Westfalian Wilhelms University Münster, Münster, Germany

**Keywords:** myosin, Myo9b, RhoGAP, cell migration, RhoA, Rac1, HL-60 cells, macrophages, actin, lamellipodia, DIC, differential interface contrast, FCS, fetal bovine serum, GAP, GTPase-activating protein, GEF, guanine nucleotide exchange factor

## Abstract

To migrate, cells assume a polarized morphology, extending forward with a leading edge with their trailing edge retracting back toward the cell body. Both cell extension and retraction critically depend on the organization and dynamics of the actin cytoskeleton, and the small, monomeric GTPases Rac and Rho are important regulators of actin. Activation of Rac induces actin polymerization and cell extension, whereas activation of Rho enhances acto-myosin II contractility and cell retraction. To coordinate migration, these processes must be carefully regulated. The myosin Myo9b, a Rho GTPase-activating protein (GAP), negatively regulates Rho activity and deletion of Myo9b in leukocytes impairs cell migration through increased Rho activity. However, it is not known whether cell motility is regulated by global or local inhibition of Rho activity by Myo9b. Here, we addressed this question by using Myo9b-deficient macrophage-like cells that expressed different recombinant Myo9b constructs. We found that Myo9b accumulates in lamellipodial extensions generated by Rac-induced actin polymerization as a function of its motor activity. Deletion of Myo9b in HL-60–derived macrophages altered cell morphology and impaired cell migration. Reintroduction of Myo9b or Myo9b motor and GAP mutants revealed that local GAP activity rescues cell morphology and migration. In summary, Rac activation leads to actin polymerization and recruitment of Myo9b, which locally inhibits Rho activity to enhance directional cell migration.

Cell function is tightly coupled with cell morphology, and coordinated alterations in cell morphology are a prerequisite for cell translocation. Cell migration depends on a polarized morphology with a protruding front and a retracting back (1, 2). Key regulators of these two opposing activities are small monomeric GTPases of the Rho subfamily (3). They regulate the dynamics and organization of the actin cytoskeleton. Rho proteins are activated by guanine nucleotide exchange factors (GEFs) that catalyze the exchange of GDP for GTP and inactivated by GTPase-activating proteins (GAPs) that accelerate GTP hydrolysis, switching the GTPase back to the inactive GDP-bound state. A third class of proteins, named GDP dissociation inhibitors, is sequestering the GTPases in the cytosol. Activation of RhoA increases filamentous actin concentration and myosin II contractility (3). Acto-myosin II contractility at the sides and back of migrating cells pushes the nucleus forward and retracts the rear (4, 5, 6). Cell protrusion is mainly driven by Rac- and Cdc42-induced actin polymerization (7). However, FRET biosensors monitoring RhoA activity indicated an enhanced RhoA activity at the protruding membrane, but the functional significance of this finding is not understood (8, 9, 10, 11).

Regulatory networks capable of self-organizing cell polarization have been identified, including positive feedback mechanisms, mutual inhibition, and inhibition with positive feedback (12). The signaling networks regulating the front and back have also been shown to reinforce each other (13, 14). To understand cell migration on a molecular level, the molecules controlling subcellular RhoA signaling circuits and their linkage with Rac1 signaling need to be determined. Many regulators of Rho and Rac signaling, as well as effector molecules, have been identified. The task is now to assign cellular functions to all of them and to integrate them into physiological circuits.

The RhoGAP myosin IXb (Myo9b) accumulates in protrusive cellular structures containing dynamic actin filaments owing to its actin-based motor activity (15). Deletion of Myo9b causes impaired cell migration both *in vitro* and *in vivo* (16, 17, 18, 19, 20). We hypothesized that Myo9b is recruited to extending lamellipodia through Rac-induced actin polymerization to locally inhibit RhoA activity at the leading edge. Local inhibition of RhoA activity by Rac activity could prevent contractility and stabilize a positive feedback loop supporting protrusion. To address this hypothesis that Myo9b acts locally, we firstly tested whether Rac activation is sufficient for the recruitment of Myo9b to protruding lamellipodia. Secondly, in HL-60 macrophages, we replaced endogenous Myo9b with Myo9b mutants lacking either GAP or motor activity and subsequently characterized the motility of these genetically modified cells. We show here that Rac activity is sufficient for Myo9b recruitment to lamellipodial protrusions and that local recruitment of Myo9b RhoGAP activity is important for directional cell migration.

## Results

### Rac activation induces Myo9b recruitment to protruding lamellipodia

**Figure 1:**
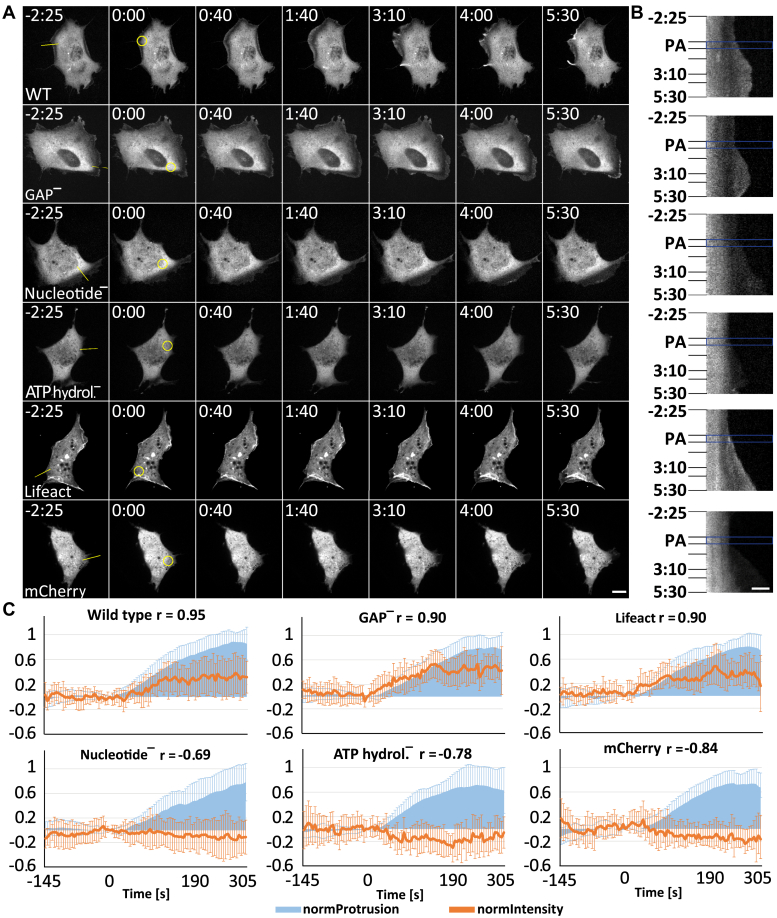
**Myo9b accumulates at the leading edge of Rac-induced protrusions.***A*–*B*, NIH/3T3 cells coexpressing PA-Rac and mCherry-Myo9b (WT), mCherry-Myo9bR1695M (GAP^−^), mCherry-Myo9bG244R (nucleotide^−^), mCherry-Myo9bR295C (ATP hydrol.^−^), Lifeact-mRFPruby, or mCherry were analyzed by live cell fluorescence microscopy. A time-lapse movie monitoring the distribution of Myo9b and control constructs was recorded with a frame rate of 5 s on a spinning disc microscope. Starting after 30 frames (145 s), PA-Rac was activated in the indicated ROI (*A*, *yellow circle* in the second images from the left) by irradiation with 405-nm light using a FRAP module. Photoactivation was repeated every 5 s for a total of 35 s, and the reaction of the cell was monitored until several minutes afterward. *A*, single images from a characteristic time-lapse movie. Time stamp indicates the time relative to photoactivation onset. The cell forms a protrusion in response to the photoactivation that persists for several minutes after photoactivation. Myo9bWT and Myo9bGAP^−^ accumulated at the leading edge of this protrusion. Myo9bWT accumulated additionally in an actin comet tail. Scale bar, 15 μm. *B*, kymographs of the movies to the left in *A* recorded along the line indicated in *yellow* in the first image of *A*. PA (*blue rectangle*) indicates the photoactivation phase. Time points of the single images shown in *A* are indicated to the left. Scale bar, 5 μm. *C*, averaged normalized protrusions (*light blue*) and corresponding construct intensities at the leading edge (*brown lines*) are plotted. Time 0 indicates the start of Rac photoactivation. For each construct, n = 10 independent photoactivation experiments. Error bars represent standard deviations. Wild type: mCherry-Myo9b wild type; GAP^−^: mCherry-Myo9b R1695M (GAP-inactive mutant); Lifeact: Lifeact-mRFPruby; Nucleotide^−^: mCherry-Myo9b G244R (motor mutant); ATP hydrol.^−^: mCherry-Myo9b R295C (motor mutant); mCherry: mCherry-tag only.

Myo9b motor activity directs Myo9b to dynamic actin filament networks that drive lamellipodial protrusion (15). How Myo9b motor activity and subsequent accumulation in protruding lamellipodia is regulated is not known. To test if Rac-induced signaling and ensuing actin polymerization would be sufficient for the recruitment of Myo9b, we transfected NIH3T3 cells with photoactivatable Rac1 (PA-Rac1). Local photoactivation of PA-Rac1 induced protrusive lamellipodia (Fig. 1, *A*–*C*). Cells cotransfected with either mCherry-Myo9b-WT or mCherry-Myo9b-R1695M, a mutant lacking RhoGAP activity, showed that both constructs accumulate at the front of those protruding lamellipodia (Fig. 1, *A*–*C*). These Myo9b constructs localized similarly to the dynamic actin filaments that drive lamellipodia protrusion as monitored by Lifeact-mRFPruby. The recruitment of Myo9b to the leading edge required its motor activity. Two motor mutants that are defective in either nucleotide binding (21) or hydrolysis (22, 23) were not recruited to the leading edge of protruding lamellipodia and localized comparable to the cytosolic protein mCherry (Fig. 1, *A*–*C*). These results show that Rac-induced lamellipodia formation leads to the recruitment of Myo9b and that its motor activity is essential for this purpose.

### Myo9b regulates HL-60 macrophage morphology and migration

**Figure 2:**
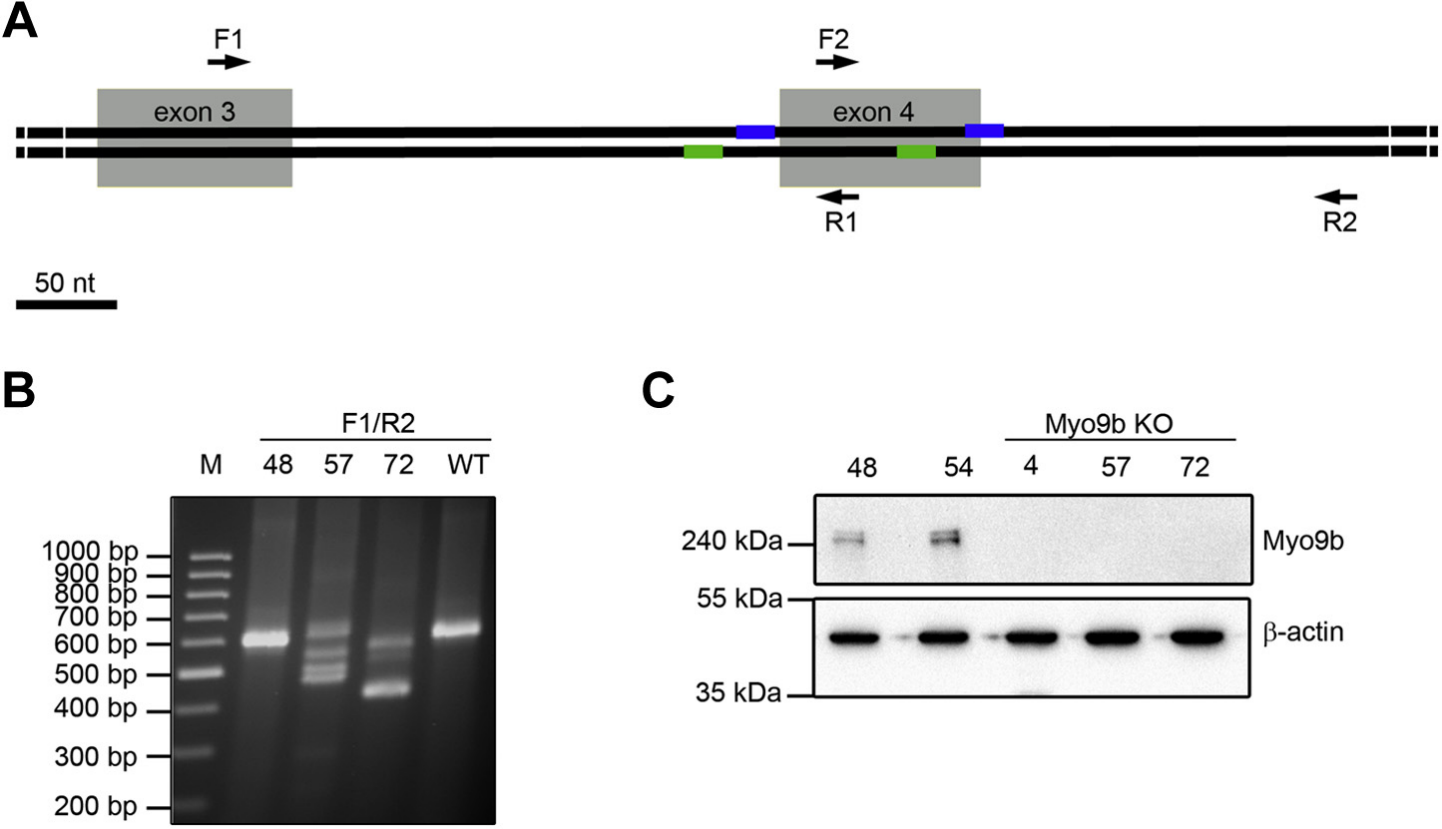
**Generation of HL-60 Myo9b knockout cell lines.***A*, schematic location of human Myo9b gene–specific gRNAs (target sites on 5′- and 3′-strand indicated as *blue* and *green lines*, respectively; *grey boxes* indicate exons) that were used together with Cas9 nickase. The aim was to induce either one or two double-stranded breaks, deleting a genomic region of ∼100 nt. *Arrows* indicate the location of primers for genotyping (F1, R2, R1, R2). *B*, PCR screening of cell clones for deletions in the Myo9b alleles. Clones 57 and 72 demonstrate deletions and insertions in the Myo9b alleles, whereas clone 48 is wild type–like. PCR was performed with primer combination F1/R2. Using genomic DNA from wild-type cells, this primer pair leads to the amplification of a 584-bp fragment. It appears that after isolation of the initial CRISPR/Cas9/GFP-expressing cell of clone 57, as monitored by GFP expression, the Myo9b alleles were further modified by CRISPR/Cas9 in a daughter cell, giving rise to more than two different Myo9b alleles in this clone. *C*, analysis of Myo9b protein expression by immunoblot. Homogenates from the indicated HL-60 cell clones were probed with Myo9b antibody. β-Actin served as a loading control. HL-60 cell clones 4, 57, and 72 are deficient for Myo9b expression (KO), while clones 48 and 54 are wild type–like.

**Figure 3:**
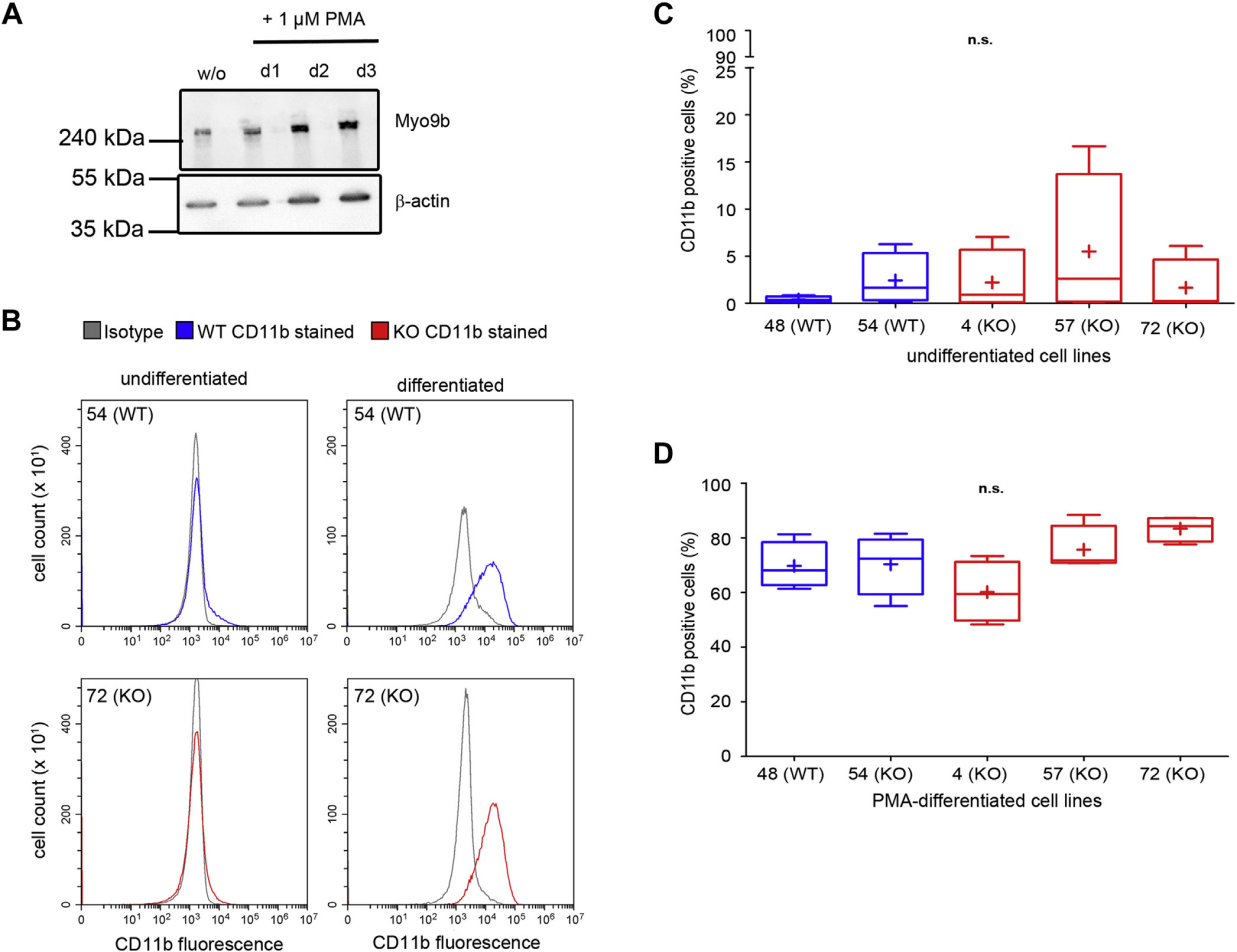
**Myo9b does not influence HL-60 cell differentiation to macrophages.***A*, Myo9b protein expression increases slightly during PMA-induced HL-60 macrophage differentiation (w/o: undifferentiated). *B*, flow cytometry analysis of the cell surface expression of differentiation marker CD11b in undifferentiated and differentiated HL-60 cells. No differences in the upregulation of expression were detected between wild-type and Myo9b-deficient cells. Histograms of a representative experiment are shown for a wild-type and a Myo9b-deficient cell line. *C*–*D*, quantification of CD11b-positive cells of indicated cell lines that were either undifferentiated (*C*) or differentiated with PMA for 1 day (*D*). Data are from four independent experiments, n.s., not significant by the Kruskal–Wallis test.

**Figure 4:**
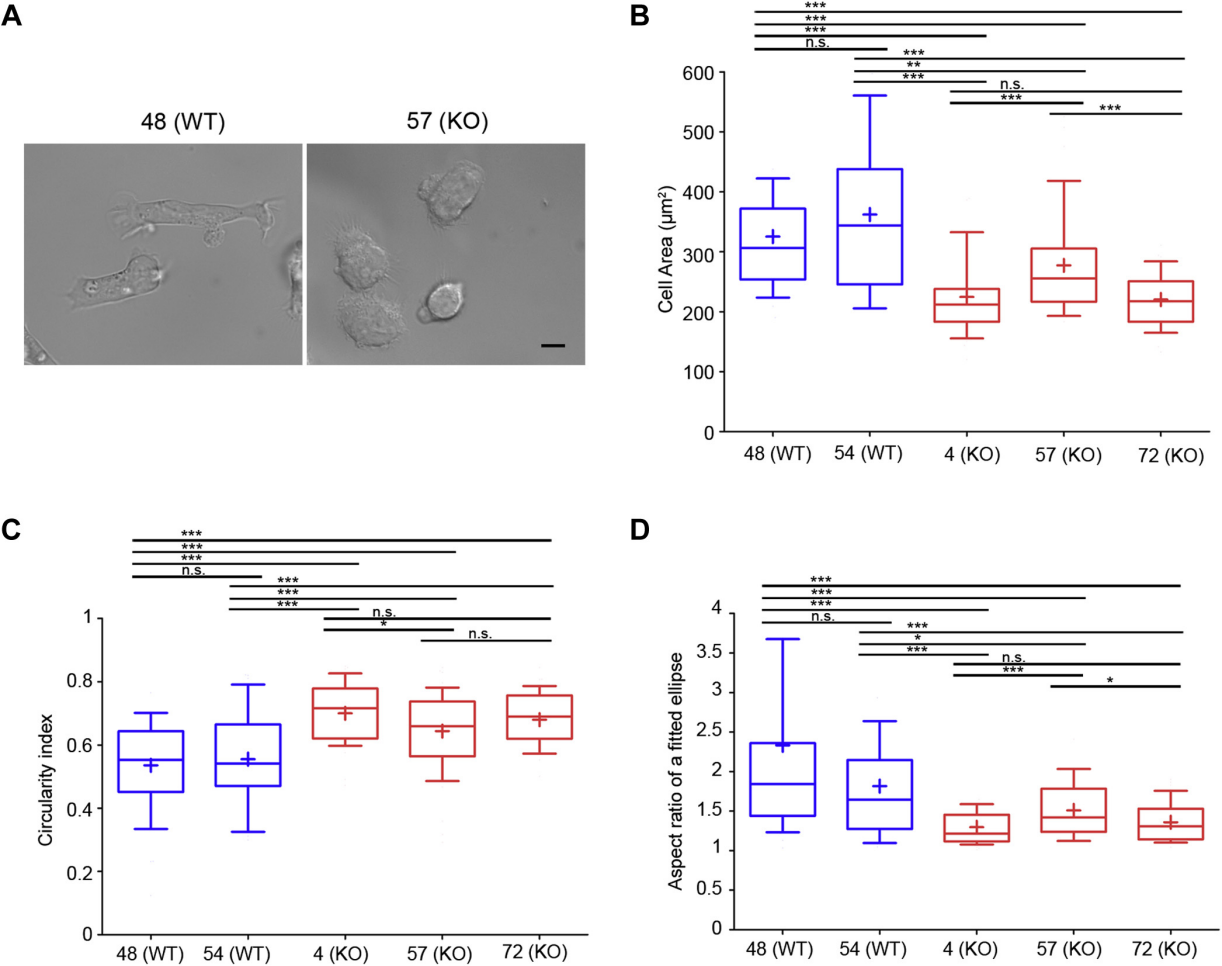
**Loss of Myo9b alters HL-60 macrophage cell morphology.***A*, HL-60 cells differentiated to macrophages in fibronectin-coated plates display a different cell morphology subject to whether they express Myo9b (48 (WT)) or not (57 (KO)). Scale bar, 20 μm. Detailed analysis of cells revealed that the Myo9b-deficient cells not only cover a smaller surface area (*B*) but also are less well polarized as indicated by an increased circularity index (*C*) and a lower aspect ratio of a filled ellipse (*D*). n = 17 to 20 cells per cell clone. A *p*-value of *p* ≥ 0.05 was regarded as not significant (n.s.), *p* ≤ 0.05 as a trend (∗), *p* ≤ 0.01 as significant (∗∗), and *p* ≤ 0.001 as highly significant (∗∗∗).

Myeloid HL-60 cells express Myo9b and can be differentiated into macrophages (Fig. 2*C* and Fig. 3). In contrast to primary macrophages, they can be genetically modified and subsequently propagated. To study the function of Myo9b in HL-60–derived macrophages, we disrupted the Myo9b alleles using CRISPR/Cas9. As schematically shown in Figure 2 A, two double-strand breaks were induced by four specific gRNA sequences and a Cas9 nickase. Selected cell clones were screened by PCR (Fig. 2*B*). The individual Myo9b alleles of potential Myo9b knockout cell clones were further analyzed by sequencing, which confirmed the insertion of nonsense mutations or deletion of nucleotides (Fig. S1). In accordance with the sequencing results, the expression of the Myo9b protein was abrogated in the corresponding cell clones as shown in Figure 2*C*. Cell clones that were not modified by CRISPR/Cas9 served as WT-like controls. WT and Myo9b-deficient HL-60 cells were differentiated into macrophages. In WT HL-60 cells Myo9b expression was not obviously altered upon cell differentiation (Fig. 3*A*). Differentiation was monitored by the expression of the marker protein CD11b. Irrespective of Myo9b expression, the differentiation marker CD11b was induced to the same extent upon HL-60 macrophage differentiation (Fig. 3, *B*–*D*). Having established that macrophage differentiation is not affected by Myo9b expression, we explored whether Myo9b regulates the morphology of adherent HL-60 macrophages. Therefore, we determined the following three parameters indicative of cell morphology: the area, the circularity index, and the aspect ratio of a fitted ellipse (Fig. 4). Cells from three different Myo9b-deficient clones covered a significantly smaller surface area than cells from WT clones, suggestive of a more contracted state (Fig. 4, *A*–*B*). This was further reflected in a higher circularity index for the Myo9b-deficient cells than in cells from WT clones (Fig. 4*C*) and a lower aspect ratio of a fitted ellipse (Fig. 4*D*).

**Figure 5:**
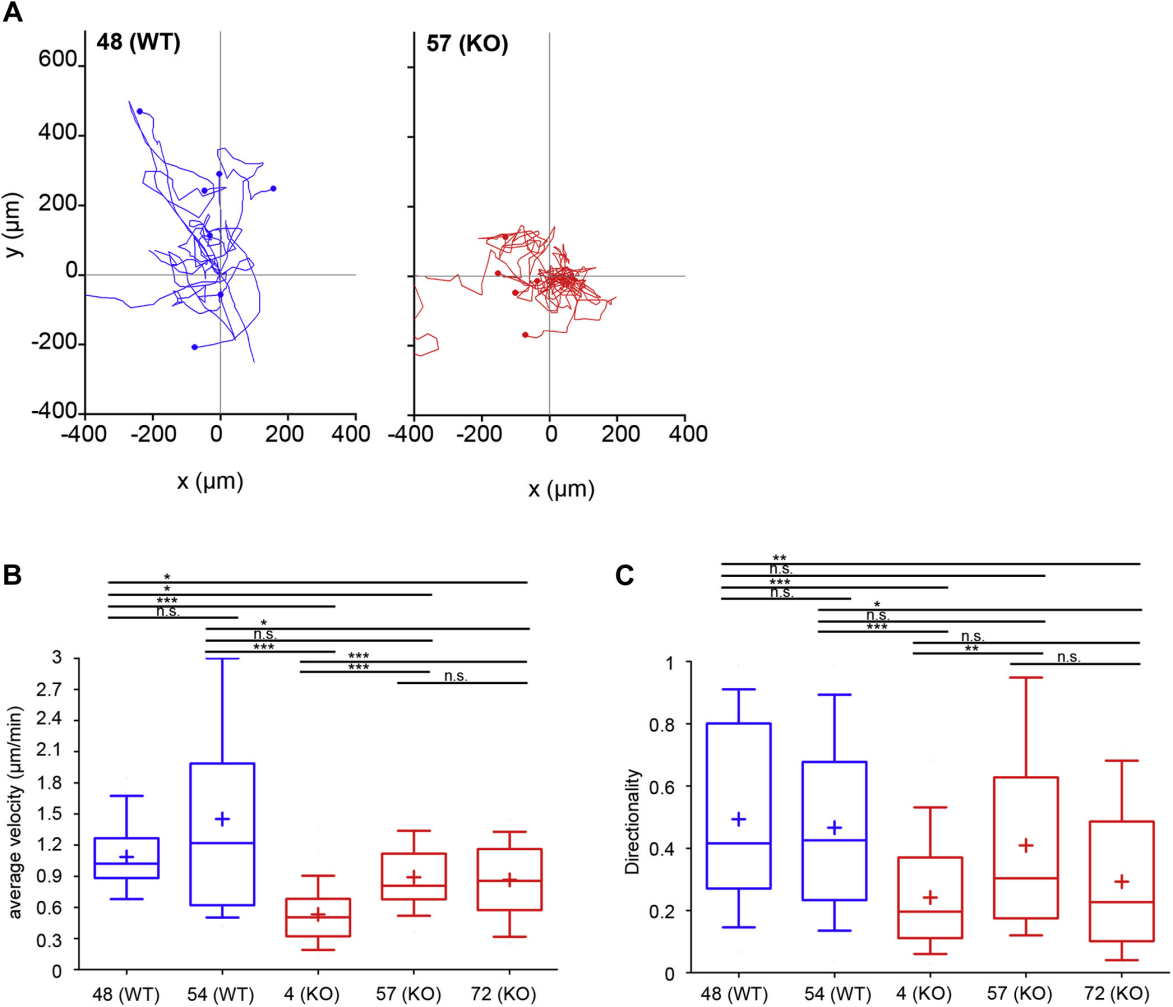
**HL-60 macrophages motility is regulated by Myo9b.** HL-60 cells differentiated to macrophages were subjected to random migration on fibronectin-coated slides. Individual cells were tracked over time (*A*) to calculate velocity (*B*) and persistence (directionality) (*C*) of cell migration. Data are from three independent experiments with 10 cells tracked each. Tracks shown in *A* were arranged to have the same origin. A *p*-value of *p* ≥ 0.05 was regarded as not significant (n.s.), *p* ≤ 0.05 as a trend (∗), *p* ≤ 0.01 as significant (∗∗), and *p* ≤ 0.001 as highly significant (∗∗∗).

Next, we tracked the two-dimensional migration of HL-60 macrophages plated on fibronectin (Fig. 5*A*). WT-like macrophages from clones 48 and 54 migrated with a faster average velocity than macrophages from the three different Myo9b-deficient clones 4, 57, and 72 (Fig. 5*B*). Analysis of the directionality of migration revealed some variability between Myo9b-deficient clones. Nevertheless, macrophages from the two WT-like clones migrated significantly more directed than macrophages from the Myo9b-deficient clones 4 and 72 (Fig. 5*C*). Cells from the Myo9b-deficient clone 57 showed somewhat variable directionality, but on average, cells were still less directed than cells of the two WT-like clones (Fig. 5*C*).

### Regulation of HL-60 macrophage morphology and migration by Myo9b requires both its RhoGAP and motor activity

**Figure 6:**
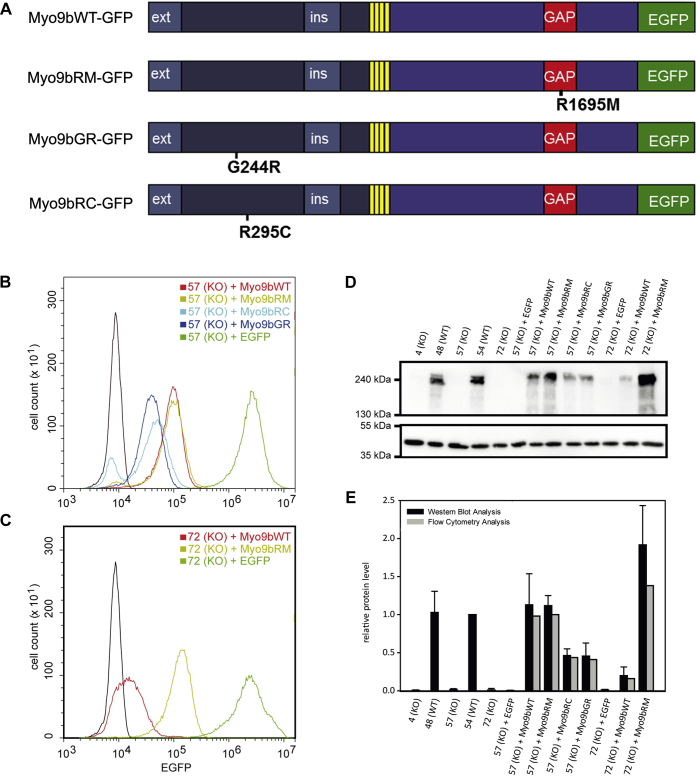
**Generation of Myo9b-deficient cell clones that express different recombinant Myo9b constructs.** Different Myo9b constructs were designed as shown schematically in *A*. Stable HL-60 cell lines were generated by lentiviral transduction of two independent Myo9b knockout clones with Myo9b WT, Myo9bGAP^−^ (Myo9bRM), and EGFP and a single Myo9b knockout clone with the two different Myo9b motor mutants nucleotide^−^ (Myo9bGR) and ATP hydrol^−^ (Myo9bRC). *B*–*C*, analysis of the expression of recombinant Myo9b-EGFP fusion constructs in cell lines derived from the Myo9b-deficient cell clone 57 (*B*) and clone 72 (*C*) by flow cytometry. *D*, an immunoblot of cell homogenates is shown demonstrating the expression of the indicated Myo9b constructs. For comparison, cell homogenates of the two wild type–like clones #48 and #54 were also probed. β-Actin served as a loading control. All recombinant Myo9b constructs had EGFP fused to their C-terminus. *E*, quantification of the expression levels of endogenous Myo9b and the different Myo9b constructs in the different cell lines. Expression was quantified using immunoblots and for the recombinant Myo9b-EGFP constructs in addition flow cytometry. Levels of Myo9b expression were normalized to either endogenous Myo9b of clone 54 or recombinant Myo9bWT in clone 57. n = 3, mean ± SD.

To test whether the altered morphology, decreased migration velocity, and directionality of Myo9b-deficient HL-60 macrophages can be rescued by the expression of Myo9b-EGFP constructs, we created cell lines of the Myo9b-deficient cell clones 57 and 72 that stably express either Myo9bWT-EGFP or just EGFP alone as a control. Furthermore, to analyze whether the phenotypes observed in Myo9b-deficient HL-60 cells are due to global or local regulation of Rho activity by Myo9b-RhoGAP, we expressed Myo9b constructs exhibiting point mutations that abrogate either its RhoGAP activity (Myo9bRM GAP^−^-EGFP) or its motor activity (Myo9bGR nucleotide^−^-EGFP and Myo9bRC hydrolysis^−^-EGFP (Fig. 6). The expression levels of the different Myo9b-mutant constructs varied somewhat (Fig. 6, *B*–*E*). Cells derived from the Myo9b-deficient clone 57 expressed Myo9bWT-EGFP and Myo9bRM GAP^−^-EGFP to comparable levels of endogenous Myo9b in WT cells. The two different Myo9b constructs predicted to lack motor activity were expressed at roughly half the amount of the other two aforementioned constructs (Fig. 6, *B*, *D* and *E*). In contrast, cell clones derived from the Myo9b-deficient clone 72 expressed Myo9bWT-EGFP to one-quarter of the endogenous Myo9b level, whereas Myo9bRM GAP^−^-EGFP was expressed at somewhat higher amounts of endogenous Myo9b levels (Fig. 6, *C*–*E*). Further analysis of the cell clones that express Myo9b-EGFP constructs by fluorescence microscopy revealed that individual cells of a given clone expressed their respective constructs at comparable levels (Fig. S2). Furthermore, the Myo9b WT and GAP^−^-EGFP constructs were enriched at the leading edge of protruding lamellipodia, whereas the two Myo9b motor mutant constructs nucleotide^−^- and hydrolysis^−^-EGFP were not and showed the same localization as EGFP alone (Fig. S2).

**Figure 7:**
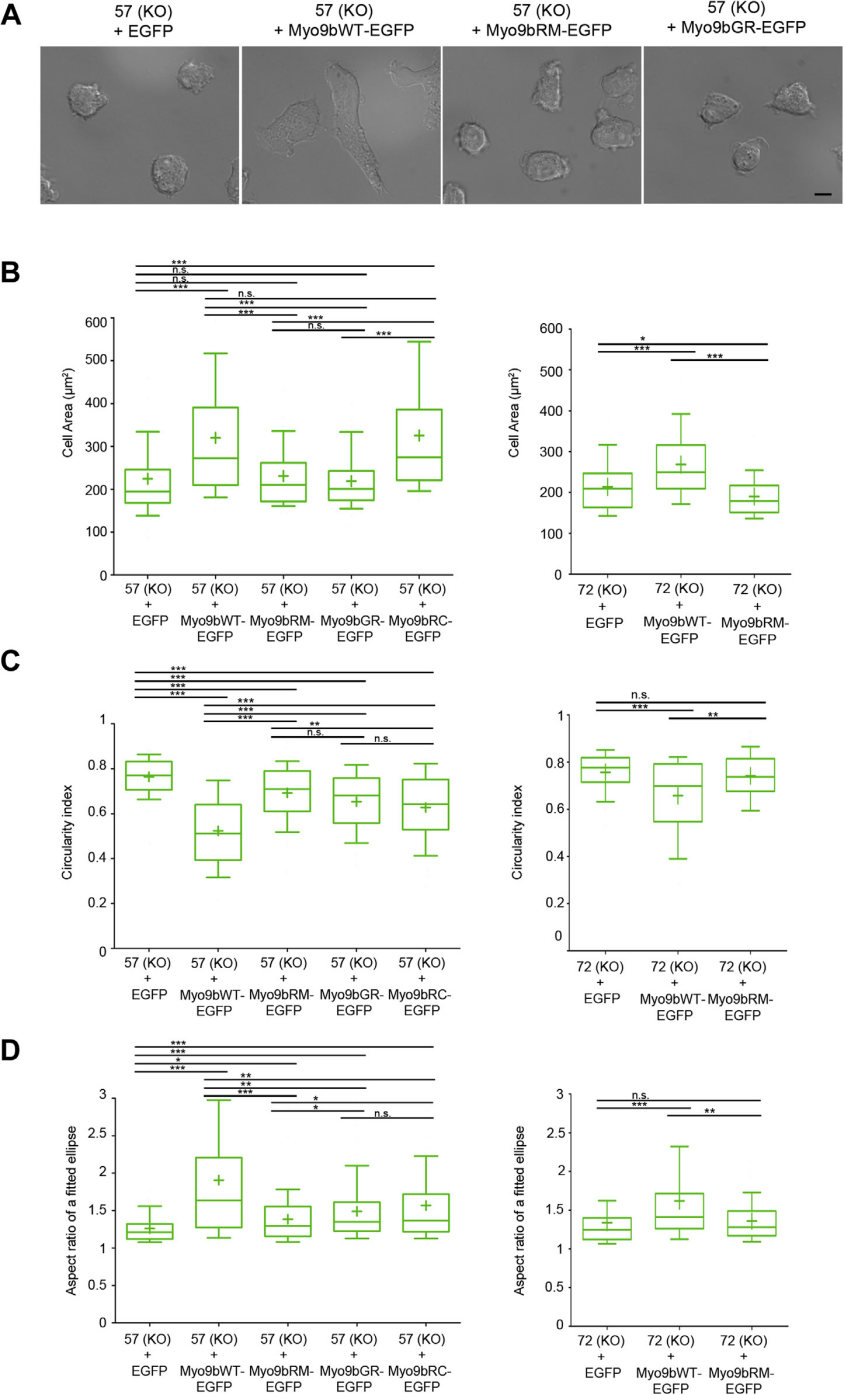
**Expression in Myo9b null HL-60 macrophages of Myo9b, but not Myo9b GAP or motor mutants, rescues cell morphology.***A*, representative DIC images of the indicated cell lines differentiated overnight with PMA to macrophages and plated on fibronectin are shown. Scale bar, 20 μm. *B*, Myo9b-deficient HL-60 macrophages expressing EGFP or the indicated Myo9b-EGFP constructs were analyzed for cell area (*B*), circularity (*C*), and aspect ratio of a fitted ellipse around the cell (*D*). Myo9b knockout clone 57– and clone 72–derived cell lines are shown. Data are from four independent experiments with 20 cells measured each. A *p*-value of *p* ≥ 0.05 was regarded as not significant (n.s.), *p* ≤ 0.05 as a trend (∗), *p* ≤ 0.01 as significant (∗∗), and *p* ≤ 0.001 as highly significant (∗∗∗).

First, we analyzed the ability of different Myo9b constructs to rescue the cell morphology of Myo9b-deficient cells adherent to a 2D surface. The expression of Myo9b-EGFP in Myo9b-deficient cells of clones 57 and 72 rescued cell spreading as shown by an increase in surface area covered by the cells, whereas cells that solely expressed EGFP still covered a smaller area (Fig. 7, *A*–*B*). It is noteworthy that expression of one-quarter amount of the endogenous Myo9b in the cells derived from clone 72 was sufficient to rescue the cell spreading phenotype. In agreement with the assumption that the smaller surface area covered by Myo9b-deficient cells is a result of higher RhoA activity, the expression of RhoGAP-inactive Myo9bRM-EGFP did not increase cell area even when expressed at twice the endogenous Myo9b level (Fig. 7, *A*–*B*). The nucleotide-binding motor mutant Myo9bGR-EGFP that does not accumulate at the leading edge also did not increase cell area. Of note, expression of the predicted ATP hydrolysis motor mutant Myo9bRC-EGFP increased cell area similar to WT Myo9b-EGFP (Fig. 7, *A*–*B*), indicating that cell area might be affected by evenly distributed diffusible RhoGAP activity. Analysis of the circularity index revealed that expression of WT Myo9b in the Myo9b null background, but not EGFP, lowered the value substantially to a level comparable to that observed with WT cells. Myo9b RhoGAP mutant as well as both motor mutants lowered the circularity index only modestly, if at all (Fig. 7*C*). Comparable results were obtained for the aspect ratio of a fitted ellipse. It increased to levels of WT cells when WT Myo9b was expressed, but only slightly increased upon expression of Myo9b RhoGAP or motor mutants (Fig. 7*D*).

**Figure 8:**
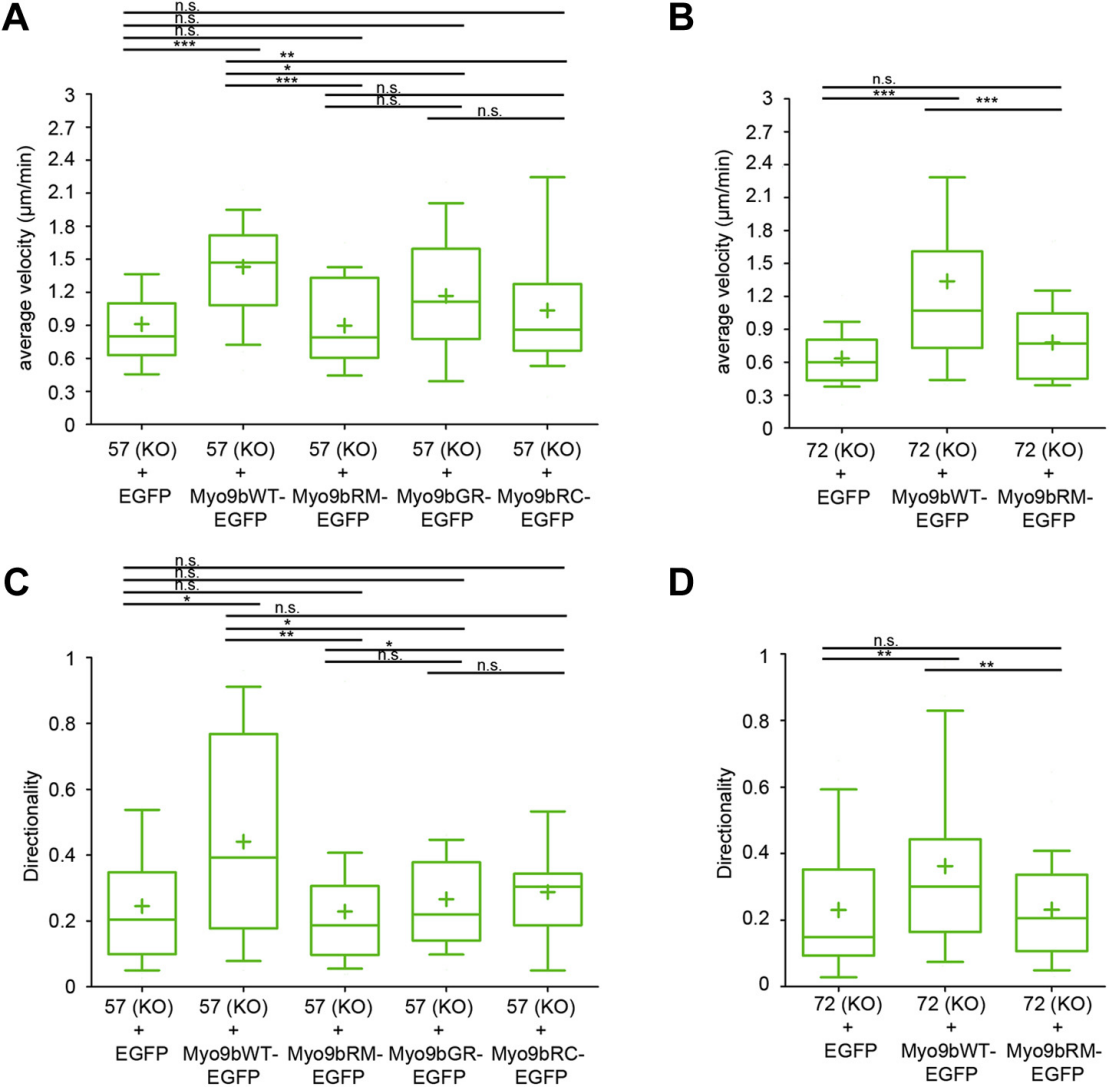
**Myo9b GAP and motor activities regulate macrophage motility.** Mutant Myo9b constructs deficient for either Myo9b GAP or Myo9b motor activity are unable to rescue migration velocity and directionality of Myo9b-deficient HL-60 macrophages. Indicated HL-60 cells differentiated to macrophages were subjected to random migration on fibronectin-coated slides, and individual cells were tracked to calculate velocity (*A*–*B*) and persistence (directionality) (*C*–*D*) of cell migration. Data are from at least three independent experiments with 10 cells tracked each. A *p*-value of *p* ≥ 0.05 was regarded as not significant (n.s.), *p* ≤ 0.05 as a trend (∗), *p* ≤ 0.01 as significant (∗∗), and *p* ≤ 0.001 as highly significant (∗∗∗).

Next, we analyzed whether the expression of different Myo9b constructs in Myo9b-deficient HL-60 macrophages affects not only their morphology but also their migration. The expression of EGFP-labeled rat Myo9b in Myo9b-deficient cells derived from either clone 57 or clone 72 increased migration velocity and directionality to WT cell levels. However, expression of EGFP did not rescue the reduced velocity and directionality (Fig. 8). Rescue of the migration phenotype depended on the RhoGAP activity of Myo9b. Expression of a RhoGAP-deficient Myo9b-EGFP mutant barely affected velocity and directionality of migration (Fig. 8). In Myo9b-deficient cells from both clones 57 and 72, velocity and directionality of migration were not significantly altered upon expression of a RhoGAP-inactive Myo9b mutant (Fig. 8, *A*–*D*). To probe for potential involvement of the motor activity of Myo9b in regulating cell migration, we analyzed the migration of cells that express either of two motor mutant constructs that are predicted to block nucleotide binding (Myo9bG244R) and ATP hydrolysis (Myo9bR295C), respectively (Fig. 6, *A*–*B*). Expression of Myo9bGR nucleotide^−^-EGFP increased neither velocity nor directionality of the cells (Fig. 8, *A* and *C*), indicating that motor activity is important. Similarly, the expression of the second Myo9bRC hydrolysis^−^-EGFP motor mutant did not yield cells that migrated faster or more directional than the original Myo9b-deficient cells (Fig. 8, *A* and *C*). These results suggest that proper positioning of the functional RhoGAP domain by the motor activity regulates cell migration and directionality.

## Discussion

Myo9b activates specifically the GTP hydrolysis of RhoA and thereby switches it to its inactive GDP-bound state (24, 25). In leucocytes, the RhoGAP activity of Myo9b regulates cell morphology and migration (16, 18, 20). But so far, it was not known whether Myo9b regulates cell morphology and migration by accelerating Rho GTPase activity in cells globally or locally. Here we show that Myo9b controls cell morphology and migration by negatively regulating Rho activity locally at sites of actin polymerization such as at the leading edge of migrating cells.

We have reported previously that Myo9b accumulates at the leading edge of protruding lamellipodia together with elongating actin filaments that drive protrusion. Prerequisite for this accumulation was a functional motor domain of Myo9b (15). Here we show that activation of Rac is sufficient to induce the relocalization of Myo9b to extending lamellipodia. Rac activation induces actin polymerization, and the motor domain of Myo9b may interact specifically with newly polymerizing actin filaments or networks. This possibility is supported by the observation that Myo9b also accumulates at filopodial tips (15). Alternatively, the Myo9b motor could be switched from an inhibited to an active state by a Rac-dependent signaling pathway that acts in parallel to actin polymerization. For instance, multiple phosphorylation sites have been identified in Myo9b (26, 27, 28, 29, 30, 31, 32, 33). However, it is currently not known whether Rac activity regulates Myo9b phosphorylation which in turn might regulate motor activity.

**Figure 9:**
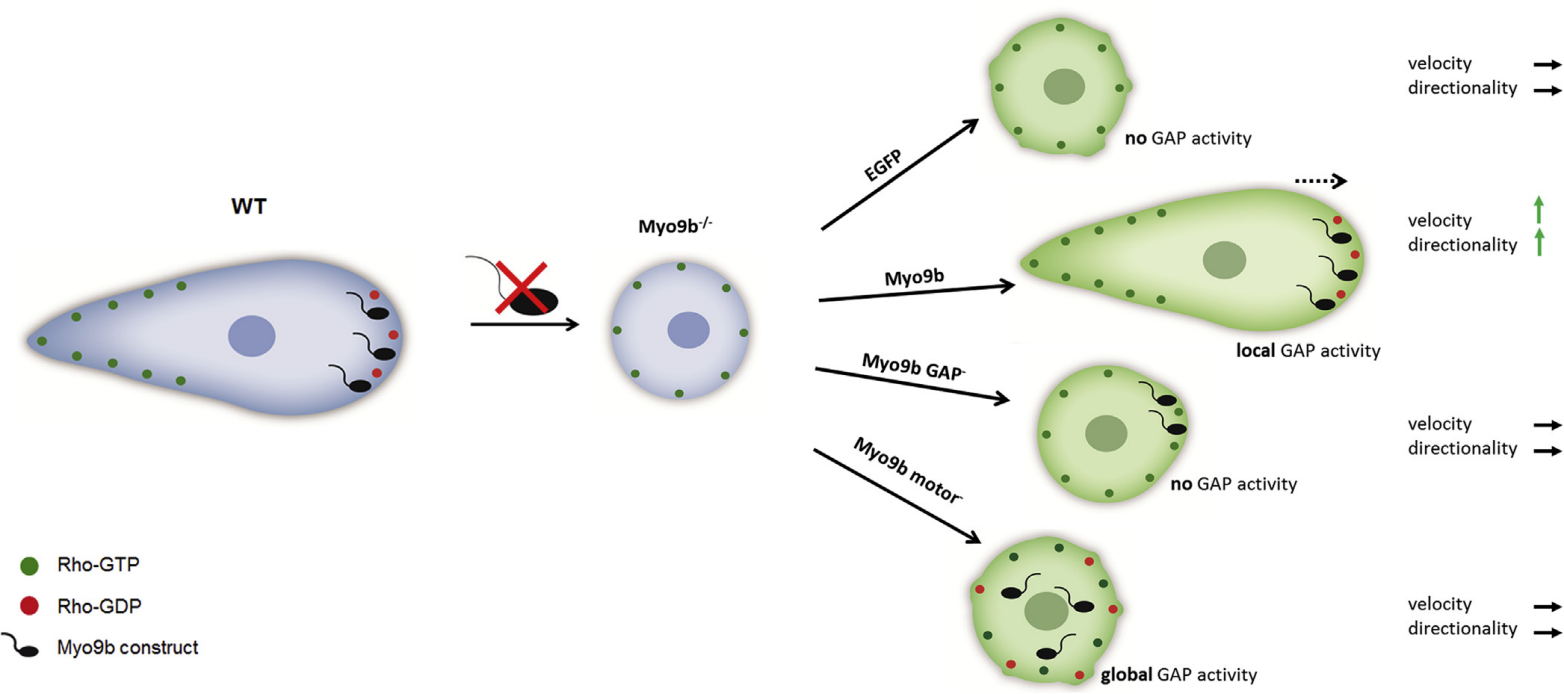
**Myo9b regulates cell migration by inhibiting RhoA activity locally at the extending cell front.** In comparison to wild-type cells, HL-60 macrophages lacking Myo9b round up and cover a smaller surface area. Furthermore, migration velocity and directionality are impaired after the loss of Myo9b. These capabilities are restored upon expression of Myo9bWT-EGFP, but not Myo9bRM-EGFP that lacks RhoGAP activity. Expression of Myo9b constructs that lack regular motor activity and are thus unable to position the RhoGAP activity properly to the extending cell front failed to rescue the migration phenotype.

As shown in the current work, to rescue the morphology and migration phenotypes of Myo9b-deficient cells, local accumulation of Myo9b in protruding lamellipodia was required in addition to Myo9b RhoGAP activity. This implies that global RhoGAP activity by cytosolic Myo9b is not sufficient to compensate for the loss of endogenous Myo9b. It was notable that levels of recombinant Myo9b as low as 20% of the endogenous Myo9b were sufficient to rescue the phenotypes. On the other hand, Myo9b motor mutants at more than twice this expression level were not able to correct the null phenotypes. Cell morphology parameters exhibited values that were somewhere in between those determined for Myo9b-deficient cells that expressed just EGFP or recombinant Myo9b WT. In contrast, cell migration velocity and directionality were not improved by the expression of the Myo9b motor mutants. These results suggest that inhibition of Rho activity globally in the cell by Myo9b slightly modifies cell morphology, but local inhibition of Rho activity by Myo9b at the leading edge is essential for cell migration (Fig. 9). We assume that the two separate missense mutations that are predicted to abrogate motor activity do not affect a potential regulation of the RhoGAP activity of Myo9b. Based on our current results, it is rather the proper subcellular positioning of the RhoGAP domain by the myosin motor that is critical for the regulation of cellular Rho signaling by Myo9b.

The spatial control of Rho signaling is critical for cell migration. Initially, it was attributed to the local activity of RhoGEFs that activate Rho at the sides and back of migrating cells (34). However, it is becoming more and more evident that RhoGAPs are participating in local regulation of Rho signaling as shown here for Myo9b. Furthermore, Myo9b mediates a cross talk between Rac and Rho signaling by inactivating Rho specifically at sites of Rac activity. This cross talk is expected to reinforce extension (frontness) and prevent contraction (backness). Indeed, it was inferred previously that protrusive F-actin locally reduces the activation of RhoA (35).

Individual cells express many different RhoGAPs, and several of those have been implicated in the regulation of cell migration (36). In the future, it will be important to investigate how Rho signaling at the cell front is coordinated and integrated by these different RhoGAP (and RhoGEF) proteins.

In conclusion, we showed that Rac activation leads to actin filament polymerization and local inhibition of Rho activity by the recruitment of the RhoGAP Myo9b. This cross talk between Rac and Rho that is mediated by Myo9b supports cell migration.

## Experimental procedures

### Plasmids

The plasmid coding for PA-Rac1 (pTriEx-mVenus-PA-Rac1; (37)) was obtained from Addgene. The plasmid coding for Lifeact-mRFPruby (38) was provided by R. Wedlich-Söldner (Münster, Germany). Plasmid pmCherry-C1 was constructed by replacing EYFP for mCherry in pEYFP-C1 using the NheI-BglII sites. The plasmids mCherry-Myo9b, mCherry-Myo9bR1695M, mCherry-Myo9bG244R, and mCherry-Myo9bR295C were constructed by substituting in the respective pEGFP-C1 constructs (15) the cDNA coding for EGFP with that of mCherry. For lentivirus production, the cDNAs coding for Myo9b, Myo9b-R1695M, Myo9b-G244R, and Myo9b-R295C were subcloned into the vector pFUGW-EGFP.

### Cell culture

Mouse embryonic fibroblast NIH/3T3 cells were cultivated in high-glucose DMEM (PAN-Biotech, Aldenbach, Germany) supplemented with 10% heat-inactivated fetal bovine serum (FCS, South African Origin Health class 1a, Cat# P30-1506, PAN-Biotech), 100 U/ml penicillin/0.1 mg/ml streptomycin (Cat# P06-07100 PAN Biotech).

HL-60 cells were a kind gift of Prof. Alexander Schmidt (ZMBE, Münster). Cells were cultured in RPMI 1640 medium (with L-Glutamine, sodium bicarbonate buffered, Cat# R8758, Sigma Aldrich) supplemented with 10% heat-inactivated fetal bovine serum, 100 U ml^−1^ penicillin/100 μg ml^−1^ streptomycin, and 1× nonessential amino acids (Cat# 11140-035, Gibco Life Technologies). They were passaged twice a week to 0.03 × 10^6^ cells/ml. Cells were diluted after 2 days by adding fresh medium to maintain cells below a density of 1 × 10^6^/ml. Differentiation toward macrophages was induced after plating 2 × 10^6^ cells/2 ml in 6-well plates by the addition of 1 μM phorbol myristate acetate (Cat# sc-3576A, Santa-Cruz Biotechnology) to HL-60 culture medium for 24 h prior to subjecting cells to experiments. All cells were maintained at 37 °C in a humidified atmosphere with 5% CO_2_.

### Transient transfection of NIH/3T3

For cotransfection, 0.04 × 10^6^ cells were plated onto a 35-mm high μ-dish (Ibidi GmbH, Martinsried, Germany) in 2 ml of culture medium and cultured overnight. SuperFect (Qiagen, Hilden, Germany) was used as a transfection reagent according to the manufacturer’s instructions. In brief, 1 μg of total plasmid DNA, consisting of 0.5 μg of PA-Rac1 and 0.5 μg of cotransfected plasmid, was diluted in 60 μl of DMEM without additives, mixed with 5 μl of transfection reagent, and incubated for 5 min at room temperature. Subsequently, 350 μl of culture medium was added to this transfection mix and added dropwise to the cells, which had previously been washed with PBS and supplemented with 600 μl of fresh culture medium. After 4 h of incubation, the supernatant was replaced by fresh culture medium and cells were incubated for another 20 h. Immediately before starting photoactivation experiments, the culture medium was exchanged to imaging medium (25 mM Hepes buffered DMEM without phenol red, Cat# 21063-029, Gibco Life Technologies, with 10% (v/v) FCS from PAN Biotech).

### CRISPR/Cas9-mediated gene knockout of human Myo9b

For CRISPR/Cas9-mediated depletion of Myo9b, four gRNAs (GTGATTTTGGCTGGAGCTGGT, GCACGCCACCCTTGCCGGCCC, GAGGAAGCCCACCATCTCCA, and GCAGTAAGTGTGCGGGCTCCC) were designed for “quadruple nicking” to introduce two DNA double-stranded breaks into the Myo9b gene. Two pX335B plasmids that contain two gRNA cassettes each (together these gRNAs facilitate one double-stranded break) were constructed, the gene for the D10A Cas9 nickase and a GFP-T2A-puromycin cassette. The plasmid pX335B was a kind gift of B. Greber (Max-Planck Institute for Molecular Biomedicine, Münster) and is a modification of the original pX335 plasmid from the Zhang Lab (39, 40). Design of gRNAs followed the online tool from the Zhang Lab (http://crispr.mit.edu/). Oligonucleotides of gRNAs were cloned into pX335B plasmid as described previously (40). Both pX335 B vectors were cotransfected using Amaxa Nucleofector Kit V (Cat# VCA-1003) and a Nucleofector II Device (Lonza, Basel) according to the manufacturers’ protocol. Cells were allowed to recover from the transfection procedure, before individual EGFP-positive cells were sorted into 96-well plates using a FACSAria (BD Bioscience) with the help of M. Stehling (Max-Planck Institute for Molecular Biomedicine, Münster). Clones were further propagated, and genomic modifications verified by PCR (primers were purchased from biomers.net GmbH: F1: TTTCAGCTCAAGCAGCCTGAAG, F2: GAGGACCTGAAGCATGACTTTG, R1: CAAAGTCATGCTTCAGGTCCTC, R2: CCTAATGACCAATGTCTTGAGC) and sequencing. Successful protein depletion was verified by Western blotting using Myo9b antibody FP3F8 (41).

### Expression of EGFP-tagged Myo9b constructs by lentiviral transduction

Lentiviral particles were generated as described (42). For transduction, 0.02 × 10^6^ HL-60 cells were suspended in either 60 μl or 80 μl of culture medium without any additives and plated in 96-well plates. Subsequently, 10 μl of EGFP virus or 30 μl of Myo9b virus solution was added to each well to a final total volume of 90 μl. The plates were incubated for 1 h at 37 °C, 5% CO_2_, before virus-treated cells were carefully transferred to 2 ml of culture medium in 6-well plates. The next day, the culture medium was replaced by spinning the cells down and resuspending them in fresh medium. Successful transduction was verified after propagating the cells for at least 48 h using a confocal microscope. Subsequently, EGFP-positive cells were selected and pooled by fluorescence-activated cell sorting.

### Rac1 photoactivation and fluorescence microscopy

Rac1 photoactivation experiments were performed with NIH/3T3 cells on an Eclipse Ti microscope (Nikon) equipped with an Ultraview VoX spinning disk setup and Modular Laser System 2.0 with 405-nm, 488-nm, and 561-nm lasers from Perkin Elmer, an Okolab H101-Priorstage top heating system, an Apo TIRF 60×/1.49 NA objective, a FRAP module, and a 14 bit C9100-50 EMCCD camera (Hamamatsu).

Transfected cells were visualized using the 561-nm laser to avoid activation of PA-Rac. A cell’s expression of PA-Rac was first confirmed by a single image illuminated with 488 nm at low intensity. Then a circular photoactivation ROI with a diameter of 10 μm was placed at the cell edge next to the protruding part of the membrane or, in cells with several protrusions, at the smallest of the protrusions. The cell’s reaction toward Rac1 photoactivation and accompanying distribution of the overexpressed proteins were observed with the 561-nm laser with an image interval of 5 s. Before photoactivation, the normal cell behavior was monitored for 30 images. Photoactivation was then performed with the FRAP function of the microscope: The ROI was exposed to 3 cycles of the 405-nm laser at 15% intensity. To observe the effect of transient Rac1 activation, cells underwent 8 of these photoactivation events with intervals of 5 s; their reaction was monitored during photoactivation and for several minutes afterward.

Rac1 photoactivation videos were analyzed using ImageJ. Kymographs of the Rac1-induced protrusions were generated using the kymograph plugin (https://www.embl.de/eamnet/html/body_kymograph.html) written by J. Rietdorf (FMI Basel, Switzerland) and A. Seitz (EMBL Heidelberg, Germany). The line of interest was set manually with the straight line tool with a line width of 1 px. In the kymographs, the line of interest is represented on the x-axis and time on the y-axis.

To analyze Myo9b enrichment at the leading edge over time, changes in average intensity were quantified over a width of 2 μm perpendicular to the cell boundary. To this end, the cell boundary in the kymographs was marked with the freehand selection tool and the area outside the cell was cleared. The boundary line was then shifted by 8 pixels (2 μm) along the x-axis, and the remainder of the cell was also cleared. Average fluorescence intensity was then determined for each time point using plot profile. To deduce coordinates of the cell boundary at each time point, a line was drawn with the freehand line tool. The following formulas were used to determine normalized intensities and protrusions:

$$I{\left(t\right)}_{norm}=\frac{I\left(t\right)-I_{\min}}{I_{\max}-I_{\min}}-\frac{I_{\left(t=0\right)}-I_{\min}}{I_{\max}-I_{\min}}$$

$$\mathrm{Pr}ot{\left(t\right)}_{norm}=\frac{{\bar{x}}_{kym}-{\bar{x}}_{kym}\min}{{\bar{x}}_{kym}\max-{\bar{x}}_{kym}\min}-\frac{{\bar{x}}_{kym}\left(t=0\right)-{\bar{x}}_{kym}\min}{{\bar{x}}_{kym}\max-{\bar{x}}_{kym}\min}$$

Data were normalized between 0 and 1 and set to zero for the time point of the start of photo activation. For every construct, kymographs of ten individual cells were exploited and the data were pooled. Plots show the averaged values with standard deviation.

### Migration assay with HL-60 macrophages

HL-60 cells were differentiated to macrophages in HL-60 medium containing 1 μM phorbol myristate acetate for 24 h on fibronectin-precoated μ-slides I (Cat# 80102, ibidi). Differentiation medium and undifferentiated cells in suspension were removed by washing twice with migration medium (RPMI 1640, 20 mM Hepes, 10% FCS) before fresh migration medium was applied for microscopy. Movies were obtained with an Axiovert 200 M Zeiss microscope using differential interface contrast (DIC) mode and a Plan-Neofluar 40×/1.3 oil immersion objective. Movies were 4 h long, and cells were imaged every 5 min, resulting in 49 frames. Cells were manually tracked using ImageJ, and random migration velocity (μm/min) and directionality were calculated and plotted. Directionality denotes the ratio of the linear distance (Euclidean distance, d_i, euclid_) from the starting point to the end point and the total distance traversed by the cell (accumulated distance, d_i, accum_).

### Morphometric analysis of HL-60 cells

Different morphometric parameters were determined for HL-60 macrophages. For this purpose, DIC images (the first frame of the movies) were analyzed using ImageJ software. The outline of individual cells was manually traced and used to calculate cell area and the cell shape descriptors circularity and aspect ratio for 17 to 20 cells per experiment. The circularity is a measure of the fit of the cell boundary to a circle with a value of one indicating a perfect circle (circularity = 4π × (Area)/(Perimeter)^2^). A continuously lower value approaching zero indicates an increasingly elongated cell shape.

The aspect ratio is a measure of how well an ellipse (*i.e.*, the ratio of its major and minor axis length) is fitted to the shape of the region of interest.

### SDS-PAGE and immunoblot

To prepare cell homogenates, adherent cells were washed twice in cold PBS before being scraped from tissue culture plates in 2 ml of PBS and being transferred to 2-ml centrifugation tubes. Suspension cells were directly transferred to centrifugation tubes and washed twice with cold PBS by centrifugation (1.5 rcf, 4 °C, 5 min). Afterward, cells were pelleted and resuspended in cell homogenization buffer (50 mM Tris/HCl pH 7.4, 100 mM NaCl, 2 mM MgCl_2_, 1% (v/v) NP-40, 10% (v/v) glycerol, 1 mM DTT, 10 μg/ml leupeptin, 10 μg/ml aprotinin, and 10 μg/ml pefabloc). Using BSA as a standard, protein concentrations of the homogenates were determined by Bradford assay. Samples were mixed with 5× Laemmli sample buffer (0.1 M Tris/HCl pH 6.8, 5 mM EDTA, 40% (w/v) sucrose, 15% (w/v) SDS, 10% (v/v) β-mercaptoethanol, 0.02% bromphenol blue) to a final concentration of 1× sample buffer before being boiled at 100 °C for 5 min. Proteins were separated on 6 to 15% acrylamide gradient gels and electrophoretically transferred to polyvinylidene difluoride membranes. Membranes were blocked in 5% (w/v) nonfat dry milk/TBST (10 mM Tris/HCl pH 7,5, 100 mM NaCl, 0,1% (v/v) Tween-20) or 5% (w/v) BSA/TBST. Blocking was followed by incubation of the membrane with the primary antibody FP3F8 mouse monoclonal Myo9b antibody (41), cell culture supernatant 1:5; ß-actin antibody clone AC-15 (Cat# A5441, Sigma), 1:2500, at RT for 1 h or 4 °C overnight. Unbound antibody was removed by washing thrice with TBST for 10 min each, before HRP-conjugated secondary antibody (Cat# 115035003, Jackson) diluted 1:5000 in TBST was added for 1 h at RT. Membranes were washed two times in TBST and once in TBS (10 mM Tris/HCl pH 7.5, 100 mM NaCl) before they were incubated with Super Signal West Pico Substrate (Cat# 34078, Thermo Fisher) and chemiluminescent signal was detected.

Expression of Myo9b and β-actin proteins was quantified by densitometry using ImageJ software. Myo9b signals were quantified relative to β-actin and normalized to WT protein level (#54). Different amounts of WT cell homogenates were loaded to verify linearity of the signals. For every cell line, results of three individual Western blots were averaged and the standard deviation was calculated.

### Flow cytometry

Staining was performed at 4 °C if not stated otherwise; 1 × 10^6^ cells were harvested per sample, transferred to 1.5-ml centrifugation tubes, pelleted at 1.8 rcf for 5 min, and washed by adding 1 ml of ice-cold PBS. To be able to eliminate dead cells from the measurements, cells were stained with ZOMBIE-NIR Dye (Cat# 423105, BioLegend). The dye was diluted in protein-free PBS 1:100 in a volume of 100 μl per sample. Cells were incubated at RT in the dark for 15 min before they were washed in flow cytometry buffer (FC buffer: 2% FCS/PBS). Subsequently, Fc receptors on the cell surface were blocked by incubation of cells with Human BD Fc Block (Cat# 564220, 0.5 mg/ml, BD Pharmingen) and diluted 1:200 in FC buffer for 15 min on ice, and then cells were washed again as described above. Fluorophore-conjugated antibodies were diluted 1:200 (anti-human CD11b-APC clone ICRF44 Cat#301310, APC-Mouse IgG1, κ Isotype control, clone MOPC-21 Cat#400120, BioLegend) in 45 μl of FC buffer per sample. Cells were incubated with the diluted antibodies for 15 min in the dark. Unstained cells served as control. Cells were washed in FC buffer, resuspended in 200 μl of FC buffer, and stored on ice in the dark until measurement. Cell fluorescence was determined using a CytoFLEX instrument (Beckman Coulter; Configuration B3-R2-V0 with two lasers, 488 nm and 638 nm) and manufacturers’ sheath fluid (Cat# B51503). The instrument was calibrated with CytoFLEX Daily QC Fluorospheres (Cat# B253230, Beckman Coulter) and operated with the CytExpert, version 1.2.11.0, software (Beckman Coulter Inc, Brea, CA). Compensation for channel spillover was determined by analyzing single-stained cells.

### Statistics

Collected data were analyzed and plotted using Prism 5 Software (GraphPad Software Inc). As Gaussian normality was rejected by the Kolmogorov–Smirnov test for at least one group per experiment, nonparametric statistics was used for analysis. The Kruskal–Wallis test was applied to check for differences between multiple groups and the Mann–Whitney U test for pairwise comparisons. Data are represented as box plots with whiskers representing the 10th to 90th percentiles. Outliers are not depicted, but the mean is represented as a “+”. A *p*-value of *p* ≥ 0.05 was regarded as not significant (n.s.), *p* ≤ 0.05 as a trend (∗), *p* ≤ 0.01 as significant (∗∗), and *p* ≤ 0.001 as highly significant (∗∗∗). Plots of individual cell tracks were generated using OriginPro 2015 SR2 software (OriginLab Corporation). Stacked bar diagrams were generated using Excel (Microscoft).

## Data availability

All the data are contained in this manuscript.

## Conflict of interest

The authors declare that they have no conflicts of interest with the contents of this article.
