# Supplementary material for: Local Myo9b RhoGAP activity regulates cell motility
Source: J Biol Chem. 2020 Dec 6;296:100136. doi: 10.1074/jbc.RA120.013623 (PMC7949024; doi:10.1074/jbc.RA120.013623)
Supplement: Supplementary Figures [file mmc1.docx]

**Supporting Information**

**Local Myo9b RhoGAP activity regulates cell motility**

Sandra A. Hemkemeyer^1^, Veith Vollmer^1^, Vera Schwarz^1^, Birgit Lohmann^1^, Ulrike Honnert^1^, Muna Taha^2^, Hans-Joachim Schnittler^2^ and Martin Bähler^1*^

Supplemental Figure S1

Supplemental Figure S2

**Supplemental Figure S1**

**Supplemental Fig. S1: Nucleotide sequences of Myo9b alleles as modified by CRISPR/Cas9 in the Myo9b-deficient cell clones.** The genomic nucleotide sequences of the Myo9b WT locus and one Myo9b WT-like clone 48 (A) or the mutant alleles of cell clones 57 and 72 are aligned (B and C, respectively). The sequences highlighted in green and blue correspond to the sequences of the four gRNAs (see Figure 2 for targeting strategy). Red color highlights nucleotide mismatches, hyphens indicate nucleotide deletions.

**Supplemental Figure S2**


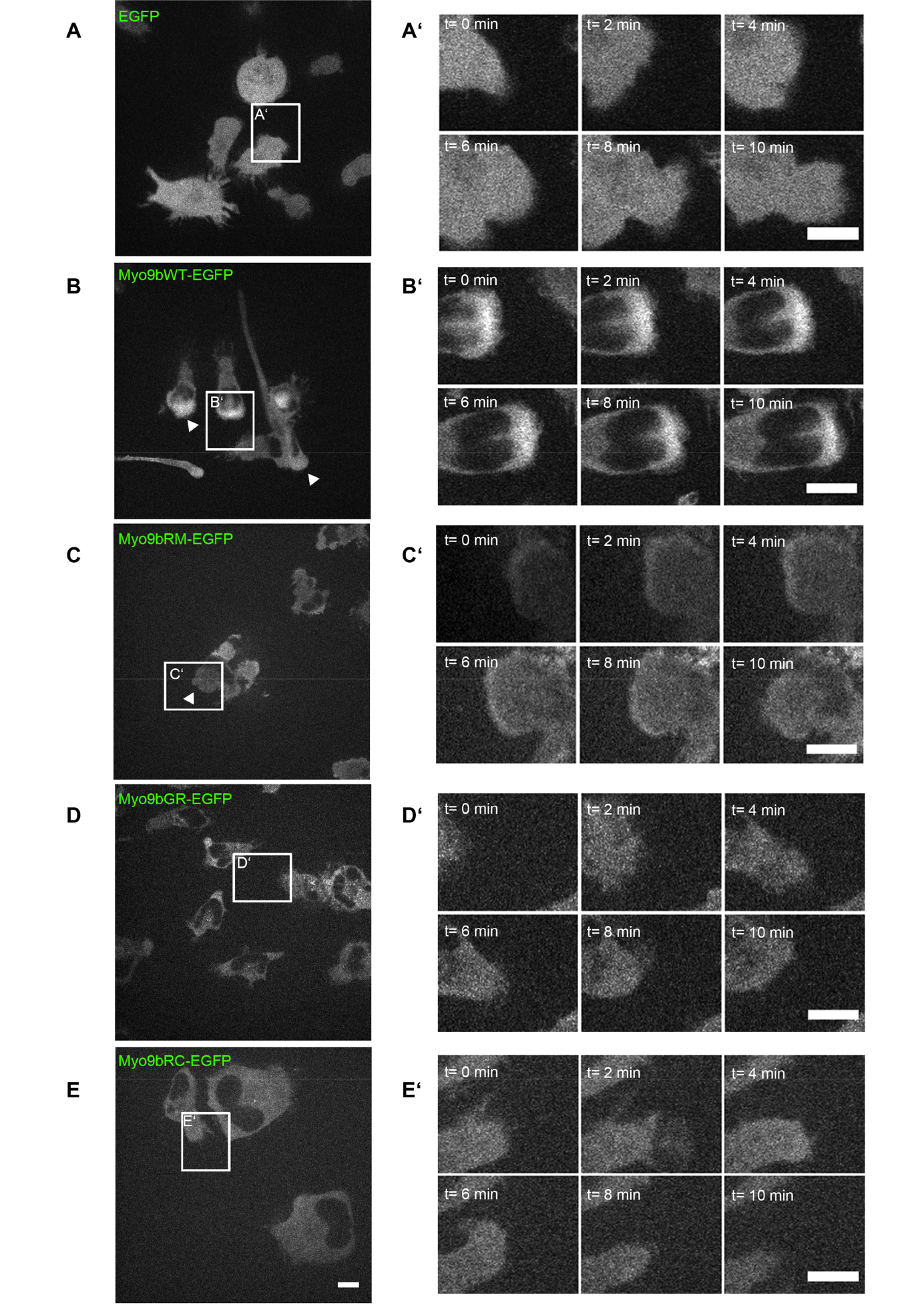


**Supplemental Fig. S2: Subcellular localization of EGFP and Myo9b-EGFP constructs expressed in Myo9b-deficient HL-60 macrophages.** Shown are fluorescence images taken from videos of differentiated Myo9b-deficient cells that stably express either EGFP (A, A’), Myo9bWT-EGFP (B, B’), Myo9bRM(GAP^-^)-EGFP (C, C’), Myo9bGR(nucleotide^-^)-EGFP (D, D’), or Myo9bRC(ATP hydrol.^-^)-EGFP (E, E’). Boxed areas in A-E) are shown enlarged at six different time points as indicated in A’-E’). Scale bars, 10 µm.
